# What are the information needs and concerns of individuals with Polycystic Kidney Disease? Results of an online survey using Facebook and social listening analysis

**DOI:** 10.1186/s12882-021-02472-1

**Published:** 2021-07-14

**Authors:** Tiffany Ma, Kelly Lambert

**Affiliations:** School of Medicine, Faculty of Science, Medicine and Health, University of Wollongong, Northfields Ave, 2522 Wollongong, NSW Australia

**Keywords:** Cross sectional study, online survey, Polycystic kidney disease, Self-management, Patient-centered care, Information needs, Patient preferences, Thematic analysis, Social listening, Diet

## Abstract

**Background:**

Polycystic Kidney Disease (PKD) is a hereditary disorder that has no cure and can result in end stage kidney failure. Searching for health information online and via social media is a common phenomenon in many medical conditions. However, no recent studies have documented the information needs, online behaviours, and concerns of people with PKD. The aim of this study was to explore the information needs of individuals with PKD and their carers by documenting (i) the information needs (ii) online information health seeking behaviours (iii) the perceived challenges of living with PKD and (iv) dietary concerns.

**Methods:**

A 17-item survey was constructed by undertaking a social listening analysis. This survey was then distributed via PKD related social media groups on Facebook. Seven groups distributed the survey with permission from the group owners. Open free text survey questions were analysed thematically using content analysis.

**Results:**

A total of 536 respondents completed the online survey (70.9 % female, 77 % aged 35–70, 70.2 % diagnosed more than 10 years ago). The major information need expressed by participants with PKD was for dietary information. Information regarding medications, medical management and symptom control were also desired. The overarching themes arising from the free text responses to the major challenge of living with PKD included ‘learning to navigate dietary ambiguities’; ‘managing social, psychological and emotional needs’; and ‘accepting an uncertain future’. In addition to a strong desire for practical and specific dietary information, participants expressed a need for more online information pertaining to management of fatigue, pain, complications and how to manage mental health. Online peer support was also highly regarded and desired.

**Conclusions:**

This study provides contemporary insights into the type of information desired by people with PKD. The results indicated that there was a strong desire for unambiguous information and guidance from health professionals to facilitate self-management, alleviate concerns, and address the complexities of living with Polycystic Kidney Disease. While diet is an important and frequently expressed need, there also remains a large demand for information on how to support psychological needs, and on medical management in order to support treatment decision making. Future work is required to develop specific, actionable and evidence-based resources for patients that are available online and through health professionals. Increased access to renal dietitians, peer support and additional training for health professionals could also improve patient-centered care and support self-management.

**Supplementary Information:**

The online version contains supplementary material available at 10.1186/s12882-021-02472-1.

## Background

PKD is a progressive, irreversible disorder characterised by the development of many cysts within the kidneys [1]. Known complications include abdominal and flank pain, hypertension, cyst rupture and infection which can lead to kidney failure, premature mortality, reduced quality of life and emotional distress [1, 2]. Partnership with health professionals is integral to the management of this complex disorder and the provision of information to patients is essential to facilitate self-management [3–5]. However, patients with PKD have previously reported a sense of confusion regarding the information provided by and available from health professionals [3]. This information has been described as ambiguous or conflicting and results in a sense of disempowerment regarding their self-management capabilities [3, 6].

The consequences that may result from inadequately meeting the information needs of patients is that patients may turn online to gain a better understanding of their condition and or treatment options [7, 8]. Online health information seeking is estimated to occur in 80 % of all patients [9], and more specifically over one third of adults use social media for health information and social support [7, 8]. Evidence also shows that 75 % of health decisions made by patients were influenced by information they received through their own research [10].

An improved understanding of the information needs and concerns of patients and carers is known to facilitate patient-centred care and improve patient-health professional relationships [5, 11–13]. However, very few studies to date have investigated the information needs of individuals living with PKD [14–16]. Therefore, the aim of this study was to explore the information needs of individuals with PKD and their carers by documenting (i) the information needs (ii) online information health seeking behaviours (iii) the perceived challenges of living with PKD and (iv) dietary concerns.

## Methods

Details of the study are reported according to the Checklist for Reporting Results of Internet.

E-Surveys (CHERRIES) [17]. This study consisted of two phases: a desk-based content analysis, followed by distribution of an online survey. Ethics approval was received from the University of Wollongong Human Research Ethics Committee for the online survey (Approval Number 2020/111). The study was conducted in accordance with the National Statement on Ethical Conduct in Human Research [18] and the conditions of ethics approval.

### Data collection of frequently asked online questions

Phase one involved in depth content analysis of frequently asked questions online. We were particularly interested in examining dietary related concerns as information obtained from Google trends (https://trends.google.com) indicated the phrase ‘polycystic kidney disease diet’ had experienced the largest increase in search frequency (+ 130 %) worldwide in the past 12 month period. To conduct the content analysis of frequently asked questions online, we utilised social listening methodology using previously established methodology [19–21]. To assist with categorisation of dietary concerns, four pre-defined search terms were entered into the Google search engine. These terms were: ‘PCKD’ OR ‘Polycystic Kidney Disease’ OR ‘PKD’ AND ‘diet’ OR ‘nutrition’ OR ‘food’ OR ‘FAQ’ (Table 1). These search terms were based on previous research analysing online renal diet information [22] and professional clinical experience. For the purpose of this content analysis all research was restricted to utilising the internet browser ‘Chrome’ and ‘Windows’ operating system, as both generate the highest site-usage compared to other browsers and operating systems (57.76 % and 68.61 %, respectively) [23]. Furthermore, the search engine ‘Google’ was chosen as this is the most frequently used search engine globally, holding 92.51 % of the global search engine market share [24]. The ‘people also ask’ function of Google was also utilised to establish common questions entered into Google relating to PKD. Results of the social listening analysis is shown in Supplementary Tables 1 and condensed in Supplementary Tables 2 and 3.

**Table 1:** Search terms used for social listening and content analysis

Polycystic kidney disease and diet	Polycystic kidney disease and nutrition	Polycystic kidney disease and food	Polycystic kidney disease and FAQ
PCKD and diet	PCKD and nutrition	PCKD and food	PCKD and FAQ
PKD and diet	PKD and nutrition	PKD and food	PKD and FAQ

#### Data collection from Facebook Groups

To evaluate frequently asked questions posted by people with PKD on Facebook, the internal search engine within Facebook was used to generate a list of active groups, based on the four previously outlined search terms in Table 1. The Facebook groups function indicated there were.

13 groups available using the search term ‘polycystic kidney disease’, 34 groups available using the search term PKD; 15 groups available using the search term. Removal of duplicates from these searches resulted in 22 groups. We chose the 5 largest groups and the Living with PKD in Australia and New Zealand groups to analyse. To conduct this content analysis, the first authors’ (TM) personal Facebook Profile was used to request membership of the group. Inclusion criteria for groups that were analysed included: groups that gave permission to be a member and groups that were in English. Exclusion criteria consisted of commercial groups, groups that were unrelated to PKD in humans, groups that included other conditions apart from PKD or private Groups where access was denied.

To undertake the Facebook content analysis, all posts made to the five largest Facebook Groups providing permission were reviewed as well as the Facebook Group titled ‘Living with PKD in Australia and New Zealand’. These groups were systematically analysed from the 6/4/2020 to 21/4/2020. All posts created within the 6-month period (30/3/2019 to 30/3/2020) or from inception of creation to 30/3/2020 were included in this study. All social media posts were reviewed manually in reverse chronological order. The posts were filtered based on ‘recent posts’ as opposed to ‘new activity’. Because of the large number of diet related Facebook posts, these posts were further categorised into: PKD and diet; PKD and nutrition: PKD and food: PKD FAQs. A summary of the posts to Facebook and are included in Supplementary 3.

#### Data collection from Online Forums for individuals with PKD

In order to identify relevant online forums, the search terms; “online forum”, “discussion board”, “discussion forum”, and “online support groups” were combined with “Polycystic Kidney Disease”, “PKD”, and “PCKD”. The online forums were screened by one member of the research team (TM) according to the title, content and purpose of the discussion. Online forums were excluded if the initial title of the online forum and content was not specifically targeted to individuals and/or carers of PKD, if the forum was designed to share advice and opinions from ‘experts’ only as opposed to allowing input from patients and/or carers [21]. The three online forums included in the content analysis were not password protected and therefore registration to view participants posts was not required.

All posts to the PKD related online forums were systematically searched during the data collection period of 2 April 2020 to 5 April 2020. To ensure that all current concerns by people with PKD were captured, all posts in online forums used the same data collection period as that used to examine Facebook posts i.e. 30/9/2019 to 30/3/2020. Only the forum titled ‘PKD Charity for Autosomal Dominant PKD’ had an archive for posts allowing this to be searched in reverse chronological order. The other two forums required a manual search of posts to identify posts that fell within the allocated 6-month period. The forum posts were also categorised using the same four predetermined search categories: ‘PKD and diet’, ‘PKD and nutrition’, ‘PKD and food’ and ‘PKD FAQ’. During this analysis no interaction was made between the researchers and the participants on the site.

#### Online survey development

The results of the content analysis of online questions, Facebook and forum posts were used to inform the design of questions in the online survey. In addition to five demographic questions, the online survey also included 12 additional questions based on the types of questions posted by people with PKD to forums, Facebook and Google ‘questions people ‘ask’. These additional questions included one free text question about the challenges of living with PKD; one free text question regarding recommendations for delivery of information to people affected by PKD; two free text questions on information and sources of information. An additional eight questions were included about the type and format of diet and nutrition information desired online. These eight questions were included based on the frequency of occurrence in the analysis of Facebook group and forum questions.A summary of questions within the survey is shown in Table 2. Face validity of the draft survey was established by obtaining feedback on the survey questions with board members of PKD Australia for peer review. These board members were individuals with PKD or carers of people with PKD. Feedback at this time indicated no changes were required. To facilitate completion of the survey by people with low health literacy, the final survey achieved a readability score of Grade 5 i.e. considered ‘fairly easy’ to read [25]. The final survey was then uploaded onto the SurveyMonkey platform. The survey did not use randomisation or adaptive questions [26] as it was determined that these would not impact responses. No identifying information or cookies were collected from participants in order to preserve confidentiality and anonymity of participants.

**Table 2:** Summary of responses rate to online survey questions (*n* = 536)

Questions	Response rate
1. Person or carer of someone with PKD	98.7 % (529/536)
2. Gender	100 % (536/536)
3. Age range	100 % (536/536)
4. Length of time since diagnosis	100 % (536/536)
5. Stage of kidney disease	97.9 % (527/536)
6. What has been the biggest challenge for you or the person living with (PKD)? (free text question)	93.7 % (502/536)
7. What type of information have you tried to find online?	96.1 % (515/536)
8. Have you used social media to find information about PKD?	93.7 % (502/536)
9. Do you follow a special diet for your PKD? (free text question)	94.6 % (507/536)
10. Have you seen a dietitian previously to get information about the diet for PKD?	97.0 % (520/536)
11. If you had a chance to ask a dietitian a question right now, what would you most like to know? (free text question)	97.9 % (525/536)
12. Would you find a cookbook for people with PKD useful?	100 % (536/536)
13. In what format would you like a cookbook?	96.6 % (518)
14. What aspects would be of interest in a cookbook ?	97.2 % (521/536)
15. Would you be interested in submitting a recipe for a cookbook ?	95.3 % (511/536)
16. Would you like to assist with compilation of a recipe book ?	98.1 % (526/536)
17. Do you have any other comment or suggestions on how to deliver nutrition information to people affected by PKD? (free text question)	48.9 % (262/536)

Legend: *PKD* Polycystic Kidney Disease

#### Survey recruitment

Participant recruitment occurred from March to May 2020 via two mechanisms. Firstly, permission was gained from administrators of eight PKD Facebook Groups utilising targeted convenience sampling [27, 28]. Secondly, snowball sampling was accomplished through email invitations sent to the PKD community from PKD Australia and the PKD Foundation (USA). Invitations to complete the study included a link to the online survey. These sampling methods were intended to facilitate access to hard-to-reach populations who may use the internet but are not social media users, and to allow data to be gathered in a reasonable length of time during the first COVID lockdown in Australia [20, 27]. Any individual with PKD (and carers) over the legal age requirements to access a Facebook and email account (13 + years) were permitted to complete the survey.

#### Survey analysis

To preserve participant confidentiality, unique site visitors were not obtained and therefore, the view rate and participation rate of the survey could not be determined. The response rate could not be accurately determined due to the changing membership size of these Facebook Groups. Nevertheless, the completion rate of the survey was recorded by Survey Monkey and completeness of each question was documented. The IP check and log file analysis were conducted and manually checked for duplicate entries. All surveys submitted were included in the analysis with incomplete questions counted as missing data. Due to the nature of the survey statistical correction was not performed.

Responses from the five open-ended survey questions were coded verbatim for analysis. Dedoose software was utilised to manage, code and categorise responses (Dedoose Version 8.3.35 2020 [29]). The Framework approach [30] guided the thematic analysis. This involved line-by-line coding of the transcript with double coding of 10 % of the free text responses by the second author (KL) to ensure agreement of codes. Refinement of codes to form categories and then amalgamation of categories to produce the final analytical framework followed. Finally, main themes were charted, and exemplar quotes were identified.

## Results

A total of 536 survey responses were received, of which 70.9 % of participants were female (Table 3). Most participants were people with PKD (92.4 %) aged 35-70yrs (77.4 %) and were diagnosed with PKD > 10yrs ago (70.2 %). Approximately one quarter of participants had early CKD (stage 1 or 2, 28.0 %, Table 3); one fifth had stage 3 CKD (22.6 %); and about one fifth were undertaking a renal replacement therapy (RRT) (21.7 %). Analysis of ISP addresses indicated that most respondents were from the United States (57.5 %), Australia (25 %) and the United Kingdom (9.9 %). Other respondents were based in Europe (2.1 %), New Zealand (1.5 %), Canada (1.5 %), South East Asia (1.5 %), Middle East (0.6 %), South or Central America (0.4 %) and Africa (0.2 %). There were no differences between respondents from the largest three groups of respondents for gender (*p* = 0.31), age (*p* = 0.08), years since diagnosis (*p* = 0.44) or stage of CKD (*p* = 0.06). Email metrics (unpublished) indicated the email was sent to 18,310 people in the US, of which 4869 people opened the email and 460 clicked on the survey link. Metrics from Australia indicated 120 people clicked on the survey from the email.

**Table 3:** Demographic characteristics of survey participants (*n* = 536)

Disease status	
The person with PKD	495 (92.4 %)
Parent of someone who has PKD	15 (2.8 %)
Carer of someone who has PKD	15 (2.8 %)
Sibling of someone with PKD	1 (0.2 %)
Friend of someone with PKD	3 (0.6 %)
Other	16 (3.0 %)
**Gender**	
Female	380 (70.9 %)
Male	156 (29.1 %)
Prefer not to say	0 (0.0 %)
**Age**	
Less than 18 years of age	12 (2.2 %)
18–34 years of age	73 (13.6 %)
35–54 years of age	238 (44.4 %)
55–70 years of age	177 (33.0 %)
70 + years of age	36 (6.7 %)
**Length of time since diagnosis of person with PKD (years)**	
Less than 1 year ago	17 (3.2 %)
1–5 years ago	79 (14.7 %)
5–10 years ago	64 (11.9 %)
More than 10 years ago	376 (70.2 %)
**Stage of PKD**	
Unsure	53 (9.9 %)
Stage 1 or 2	150 (28.0 %)
Stage 3	121 (22.6 %)
Stage 4 (predialysis)	85 (15.9 %)
Stage 5 (dialysis or have had a transplant)	116 (21.7 %)
Other comments	31 (5.8 %)
No response	1 (0.2 %)
**Location of respondent**	
USA	308 (57.5 %)
Australia	134 (25 %)
United Kingdom	53 (9.9 %)
Europe	11 (2.1 %)
New Zealand	8 (1.5 %)
Canada	8 (1.5 %)
South East Asia	8 (1.5 %)
Middle East	3 (0.6 %)
South or Central America	2 (0.4 %)
Africa	1 (0.2 %)

Legend: *PKD* Polycystic Kidney Disease. Stage of PKD had *n* = 525 responses

The results of the survey regarding online information seeking, use of social media, prevalence of following a special diet and access to a dietitian are shown in Table 4. The most frequent type of information sought online was diet or nutrition related (81 %), followed by information about diagnosis (79 %). Information to assist with symptoms was reported by 46.2 %. While 74.9 % of respondents indicated they followed a special diet, only 61.1 % had seen a dietitian previously regarding their PKD.

**Table 4:** Patient responses to survey questions regarding types and sources of information

Survey question number	Number of Participant Responses	Percentage of Participant Responses
**7. What type of information have you tried to find online?**		
Help with day to day management such as fatigue, pain and symptoms	246	46.2 %
To better understand the condition/diagnosis	420	79.0 %
To find recommendations on medication/dialysis/surgery	215	40.4 %
To find recommendations on hospitals/nephrologists	144	27.1 %
To find information about the type of diet/nutrition guidelines for PKD	431	81.0 %
Other comments	63	11.8 %
No response	4	0.7 %
**8. Have you used social media to find information on PKD?**		
Facebook	351	69.9 %
YouTube	129	25.7 %
Instagram	48	9.6 %
Pinterest	23	4.6 %
Twitter	23	4.6 %
Another social media platform	61	12.2 %
I don’t use social media	72	14.3 %
No response	34	6.3 %
**9. Do you follow a special diet for your PKD ?**		
No	97	19.6 %
Yes	371	74.9 %
Did not answer	27	5.5 %
**10. Have you seen a dietitian previously to get information about the diet for PKD?**		
Yes	193	36.1 %
No	327	61.1 %
Other comments	45	8.4 %
No response	1	0.2 %

Four major themes emerged regarding the information needs of people with PKD. These themes were: ‘A challenging journey’; ‘A need for information’; ‘Dietary concerns’; and ‘A need for practical resources’. Exemplar quotes are provided to illustrate these themes and are abbreviated to participant number to preserve confidentiality. Additional quotes are provided in Table 5.

**Table 5:** Exemplar quotes from free text questions in the online survey

**A challenging journey**
The biggest challenge is the physical pain. I stay active and continue to live my life but there are days where it is almost unbearable and severely affects my mental health
(my biggest challenge) is pressure to have children before kidney’s fail
**A need for information**
I want to know….the best diet for managing early progression of the disease … best way to implement it in order to mitigate other potential risks that might be associated with it (e.g., cardiovascular disease)?
Having a Facebook group for diet and nutrition or a website devoted to diet and nutrition for PKD would be awesome
Make information available at Nephrologist…supply new patients with links, app info, websites etc…Otherwise you are left with the internet to find the info and it may not always be reliable
Doctors…should be encouraged to make a referral to a dietician at the early stages of this disease
What the recovery of kidney transplant, along with nephrectomy, is really like
What can I do to slow down the growth of the cysts?
**Dietary concerns**
There is limited access to dietician via health insurance & doctors don’t seem to make priority for those in early stages of disease
Most dieticians will probably say low salt, and healthy diet. Not very specific
Are there foods that are less stressful on kidneys in general, or things I should avoid besides the broad low-sodium recommendation
What research is there on benefits of specific dietary components ?
(my biggest concern) is… am I doing the right thing (with my diet)? What should I do?
**A need for practical resources**
Data that’s understandable to the general public about how impactful healthy diets are for those who follow them vs. those who don’t
Simple meal plans …and recipes…according to what stage you’re in, how much protein, phosphorus and potassium
Educating nephrologists worldwide. I find myself being more knowledgeable on modern clinical trials, medicines, diet, etc. for PKD patients
Make (the information) really simple and compelling
A table or matrix that would help individuals identify what is suitable for them would be helpful

### A challenging journey

.

Living with PKD was described as a challenging, emotional and arduous journey. Participants described difficulties obtaining clear and accurate information from health professionals about symptom management and diet.

*“…It’s challenging to get accurate info re amount of potassium in foods” (Participant 169, person with PKD, 70 + years, female)*.

*“Managing my health and mental health. I feel physically as though I should be doing things that I don’t always feel like doing…I feel like this is takes an emotional toll as well as a physical toll” (Participant 371, person with PKD, 55–70 years, female)*.

Participants also described their frustrations at the vague information encountered about the course of the disease.

*“there (is) so little knowledge/ research about PKD… Will (my kidneys) be large as footballs and I cannot do anything. Or can I? Like diet” (Participant 307, person with PKD, 55–70 years, female)*.

Frustrations about the lack of specific information led many to conduct their own research online.

*"I am just learning now through the internet that a keto diet can be helpful in shrinking the cysts. My doctor has not mentioned this to me. I am trying to do more research on my own since now my 3 year old daughter has been just diagnosed a year ago” (Participant 21, person with PKD, 18–34 years, female)"*

*"Make information available at Nephrologist - so they are able to supply new patients with links, app info, websites etc at their first appointment. Otherwise you are left with the internet to find the info and it may not always be reliable (Participant 161, person with PKD, 35–54 years, male)"*

Another important challenge described by participants was learning how to live with the disease and manage the complex physical, psychological and emotional impacts. Many described frustrations at low public awareness of the disease and inadequate resources to support their care.

*“Living with regular pain and trying to do “normal” things…All with lack of understanding…because I ‘look fine’” (Participant 28, person with PKD, 55–70 years, male)*.

*“Maintaining optimum health whilst juggling work and family life (is a challenge) …while dealing with the symptoms of renal failure” (Participant 325, person with PKD, 70 + years, male)*.

Participants also expressed a range of negative emotions including fear, isolation, disappointment, loss of hope, anxiety, frustration and an emotional/psychological burden from living with PKD. Participants described living with an uncertain future as burdensome.

*“Dealing with the unknown…and getting a clear understanding of my symptoms and prognosis. There is still so much research that needs to be done” (Participant 113, person with PKD, 35–54 years, female)*.

### A need for information

There was an overwhelming consensus among participants that they desired additional information. While this information included details on medical management and social support, the most requested type of health information was for dietary information (reported by 81.0 % of survey respondents). Some of the reasons provided for requesting dietary information were to inform them of the ‘correct’ approach to manage their disease; to increase knowledge about the suitability of popular diets; and to clarify ambiguities. Recipes were also highly desirable by respondents.

*“(I want information on) the effects of the Keto diet, intermittent fasting, & other dietary options potentially beneficial to those with PKD” (Participant 6, carer of someone with PKD, 55–70 years, female)*.

Information on the medical management of PKD was reported by 79.0 % of participants This was often regarding the pathophysiology of the disease, treatment options, and life expectancy. Additional information on how to delay disease progression, the functions of the kidneys; how to interpret blood tests and symptom management were also requested.

*“I (want information on) if I will live shorter than average; survival rates; success stories (Participant 38, person with PKD, 55–70 years, female)*.

Participants also strongly desired information on how to support their psychosocial needs. Support groups either face to face or online were desired and were perceived to help provide them with reassurance, and a sense of connection and community.

*“(I want information on) support groups for patients and family of patients” (Participant 41, person with PKD, 55–70 years, female)*.

When asked about the ideal method for meeting their information needs, many participants reported they preferred the accessibility of online sources. Participants specifically expressed a desire for additional online information available through peak organisations. Facebook, including Facebook Groups, also was a highly regarded social media platform amongst participants to locate resources and connect with others.

Participants desired information about PKD from the time of diagnosis from Primary Care Physicians (PCPs) and/or Nephrologists and felt that this should be readily available to access in clinics.

*“Make information available at Nephrologist…supply new patients with links, app info, websites…Otherwise you are left with the internet to find the info and it may not always be reliable” (Participant 161, person with PKD, 35–54 years, male)*.

To ensure their dietary information needs were met, many suggested referrals to a renal Dietitian were needed, and many suggested it would ensure patients were provided with reliable, credible, and personalised dietary information and care.

### Dietary Concerns

A desire for dietary information was a strongly expressed information need and participants felt there was a lack of clear information and guidance for practical implementation of dietary modifications at each stage of PKD.

*“Different stages of PKD require changes in diet. I would specifically like to know what foods should I avoid in the different stages” (Participant 75, person with PKD, 55–70 years, female)*.

*“Are there foods that are less stressful on kidneys…or things I should avoid besides the broad low-sodium recommendation” (Participant 94, person with PKD, 35–54 years, female)*.

Information on how to support adherence to specialised renal diets was also desired.

*“I try to follow a low sodium diet…very difficult to maintain ” (Participant 294, person with PKD, 55–70 years, female)*

*"How do I know how much mg of phosphate or protein or potassium I have consumed in a day. This is quite challenging for me. (Participant 42, person with PKD, 55–70 years, female)"*.

Participants also specifically reported a desire for clarification about the dietary ambiguities they had encountered in their PKD journey:

*“What diet should I follow for PKD, there is so much conflicting information” (Participant 251, person with PKD, 18–34 years, female)*.

Clear information that is specifically designed to answer the common questions ‘what is the best diet to follow for PKD?’ and ‘what diet should be avoided for PKD?’ was highly regarded by participants.

### A need for practical resources

Dietary resources that were both informative and practical were frequently reported to be scarce but highly desirable.

*“When your (sic) diagnosed it’s very overwhelming and very difficult to find diet info…One list says you can eat this the other list says you can’t eat this…just not enough information…for PKD patients” (Participant 59, person with PKD, 35–54 years, male).*

*“… I just (want) simple meal plans…and recipes…according to what stage your in, how much protein, phosphorus and potassium…(sic)” (Participant 184, person with PKD, 18–34 years, female)*.

*“Simple is best… The hardest part of any diet is too much of a focus on what CAN’T be eaten, or recipes that are too complicated” (Participant 141, person with PKD, 35–54 years, female)*.

Participants also perceived there was a need for educational resources for health professionals. This perception arose from interactions with clinicians where they were perceived by patients or had stated outright that they had limited knowledge of PKD.

*“…educating nephrologists worldwide. I find myself being more knowledgeable…on modern clinical trials, medicines, diet, etc for PKD patients” (Participant 25, parent of person with PKD, 18–34 years, male)*

*“She was only 10months old when diagnosed, all of her close family were devastated at how we were told and the lack of knowledge GPs have about PKD (Participant 461, person with PKD, 70+ years, female)”*

*“Finding information about "living with PKD." My nephrologist monitors lab signs and progression of disease, but has little to offer about managing pain, early satiety, diet, etc (Participant 124, 35-54 years, female)”*

*“… I find myself being more knowledgeable than my current & past nephrologists on modern clinical trials, medicines, diet, etc for PKD patients (Participant 25, parent of person with PKD, 18-34 years, male)”*.

A number of survey respondents indicated that information should also be available for individuals at all stages of the disease and for a variety of ethnic backgrounds. For example, one respondent indicated there was a lack of information for children, and another stated:

*Ethnic minority patients with poor English may be hard to reach -- and may be in more need of help (and information)…(Participant 228, person with PKD, 35–54 years, female)*.

## Discussion

Information has been referred to as the ‘lifeblood of care’ [31] and many patients and carers experience a lack of information. In this study people with PKD also expressed a similar desire and strong need for additional information regarding the medical and dietary management of the disease. Participants felt confused, overwhelmed and unsupported to navigate the complexities of the management of their disease. Many also expressed feelings of anxiety relating to the unavoidable progression of the disease and uncertainty for their future. Our findings suggest that the PKD population are motivated to actively partake in their own self-management but need specific, practical information, especially pertaining to diet. Connections to the broader PKD community via social media are also considered a valuable source of psychosocial support and facilitate the provision of information to people with PKD.

The findings from this study provide a useful contribution to the limited research in this area of nephrology. Our research extends previous work which primarily used interviews, focus groups and observations from clinical consultations, therefore limiting number of participants in the studies [12, 32, 33]. The findings of this study are also similar to previous research exploring the information needs of adults with CKD more broadly. Previous research in the CKD population has consistently reported that the renal diet was difficult to comprehend, and individuals frequently encountered unclear, contradictory or inadequate advice [6, 34–36]. Like the findings in this study, previous work also describes the emotional turmoil and impact that conflicting and / or ambiguous information resulted in [34–36]. A lack of appropriate information resources to support the self-management of culturally and linguistically diverse patients with CKD is also common [37, 38].

A high proportion of respondents in this survey followed a special diet. Yet many participants in this study expressed a tangible sense of frustration and confusion because of vague and inconsistent dietary information. This was compounded by an inability to access renal nutrition expertise as well as inconsistent dietary information from primary care physicians and nephrologists. Physicians who treat people with PKD have previously reported that time constraints on patient visits were not conducive to facilitating adequate discussions and education needed [13, 38, 39]. Clinicians have also reported a lack of knowledge about how to refer to dietitians [40], and of dietetic staff shortages [40, 41].

Participants in this study used social media to search for information relating to PKD, particularly Facebook. Facebook Groups were reported as a preferred platform to find dietary information resources. This may be unsurprising given the survey was administered via this mechanism. More concerning however is the unregulated nature of many social media groups which lack oversight by scientific advisory committees. The potential for misinformation to be distributed via these forums is high. Previous work demonstrated a substantial amount of information found online for individuals with CKD is low in quality, non-evidence based, difficult to understand and hard to action [22, 42]. Unfortunately, this work is more than 5 years old and further work to evaluate accuracy, reliability, actionability and quality of online renal diet information is recommended. This will enable dietitians and other health professionals to guide patients to reliable sources of online information, including appropriate blogs and forums [34].

Like other studies [5, 35, 43], we found participants have an unmet need for disease-specific information. Previous studies have documented that primary care physicians did not feel they had sufficient knowledge about PKD [15, 38], lacked familiarity with CKD guidelines, and perceived that guidelines are unclear [15, 38]. This results in suboptimal implementation of management guidelines [15, 38]. Strategies to improve primary care physician knowledge of PKD appears warranted, particularly regarding fertility, disease prognosis and survival rates is required. This can better equip individuals in understanding the impact that PKD will have upon their future, allow them to make more informed decisions on treatment decisions, improve quality of life, and alleviate concerns regarding uncertainty of their future [5, 35, 44].

Future work should be directed at creating a standardised referral pathway for people with PKD to access renal dietitians from diagnosis of the disease. This will facilitate a more patient centred approach to self-management. However, in the United States this is complicated by Medicare reimbursement rules which only cover medical nutrition therapy for individuals with estimated glomerular filtration rates of 13 to 50 mL/min/1.73 m^2^, consistent with CKD stages G3-G5. However, most individuals with kidney disease never receive consultation from a dietitian before initiating dialysis [45]. Alternative strategies are needed to improve access to renal dietitians to clarify ambiguities, translate complex nutritional guidelines into simple and actionable dietary advice and better equip carers to support patients to change their diet [46, 47]. Programs such as the online Ren.Nu PKD Nutrition Program may help improve access to specialised credible dietetic care for people with PKD.

Creation of high-quality evidence-based resources for individuals with PKD is required. This includes resources for underrepresented groups identified in the study including individuals in the early stages of PKD, children, adolescents and individuals from culturally and linguistically diverse groups. One topic identified from the findings of this study for immediate action is development of renal diet resources for each stage of PKD outlining the dietary modifications needed and how these can contribute to good health. Similarly, creation of resources on topics such as pathophysiology of the disease; symptom management; and medical management is encouraged. The resources should be tested with the PKD community prior to release to ensure they are understandable and actionable as well as evidence based. The perspectives of individuals with PKD (and their carers) should be incorporated regarding the preferred content and format when developed as these may vary according to geography, culture and health jurisdiction [22, 48]. Investigation of how individuals with PKD utilize these resources for self-management in addition to advice received from health professionals would be useful.

Knowledge about how to access peers to provide psychosocial support has been suggested by participants in this study. In the absence of local support groups or formal networks, many people with PKD turn online. It is recommended that clinicians take a more proactive role in promoting peer relationships at all stages of the disease, and direct patients to reliable peer support groups that offer evidence-based information. Examples of such groups are the peer support program developed by the National Kidney Foundation (NFK) [49] and the Facebook Group run by the committee of PKD Australia. Increased access to peer support has potential to help individuals with PKD to adjust to living with the disease, better self-management of their symptoms, and improve their quality of life through decreased feelings of isolation [49, 50]. Another recommendation from this study is to provide more education to health professionals to improve provision of accurate and consistent information to individuals with PKD, tailored to their stage of the disease [5]. For example, the renal dietitian community could facilitate regular workshops for nephrologists and PCPs regarding dietary management of PKD similar to the ‘Nutrition Management Training Program’ [51] through NKDEP.

There are several limitations in this study. The survey was only conducted online, thus excluding individuals who may not be unable to afford access to technology or are not digitally literate. The survey was also only available in English which could result in neglecting perspectives from culturally and linguistically diverse backgrounds limiting the generalisability of findings to these groups. Due to the survey design, we may have also not adequately captured all information needs, especially those of people not active in the online PKD community. This may have resulted in skewed results toward dietary information. Despite this there are several strengths, including the large sample size, content analysis informed survey design, and in-depth qualitative analysis of the open-ended survey questions. The targeted survey also enabled an in-depth exploration of information needs and concerns of people with PKD, which was previously identified as a gap in the literature [3, 52]. The demographic characteristics of participants were similar to the general profile of people with PKD where the condition is most prevalent in females aged 50–64 years [53]. To ensure rigorous and transparent reporting the CHERRIES checklist has also been utilised [17].

## Conclusions

This study has highlighted a strong desire for unambiguous information, particularly relating to diet, and guidance from health professionals to facilitate self-management; alleviate concerns; and address complexities of living with the PKD. While dietary information is an important and frequently expressed need, there remains a demand for information on psychosocial support, pathophysiology and prognosis of the disease, symptom management, and medical management to inform treatment decisions and improve quality of life. Future work is required to develop specific, actionable and evidence-based resources utilising perspectives from such individuals on content and format. Availability of additional practical and specific resources via health professionals is encouraged to improve patient centred care and support self-management.

## Supplementary Information

**Additional file 1: Supplementary Table S1**: Results of social listening analysis.

**Additional file 2: Supplementary Table S2**. Summary of social listening analysis of frequently asked questions by individuals with PKD and their carers using Google ‘people questions ask’.

**Additional file 3: Supplementary Table S3**: Categorisation of all Facebook Groups posts.

## Data Availability

The datasets used and/or analysed during the current study are available from the corresponding author on reasonable request.
