# Supplementary material for: What are the information needs and concerns of individuals with Polycystic Kidney Disease? Results of an online survey using Facebook and social listening analysis
Source: BMC Nephrol. 2021 Jul 14;22:263. doi: 10.1186/s12882-021-02472-1 (PMC8281574; doi:10.1186/s12882-021-02472-1)
Supplement: Supplementary file 1 — Additional file 1: Supplementary Table S1: Results of social listening analysis. [file 12882_2021_2472_MOESM1_ESM.docx]

Supplementary Table 1: Results of social listening analysis

| **Search term** | **Google question initial drop down menu suggested questions** | **"People also ask" results based on Google question** | **‘Related Searches’ results related to search term** |
| --- | --- | --- | --- |
| PKD and diet | PKD and keto diet | What foods should you avoid with polycystic kidney disease? | Polycystic kidney disease diet recipes |
|  | PKD and vegan diet | What should I eat if I have PKD? | What foods should you avoid with polycystic kidney disease? |
|  | PKD and alkaline diet | What is the life expectancy of someone with polycystic kidney disease? | polycystic kidney disease in children |
|  | PKD diet chart | How is PKD passed down? | polycystic kidney disease treatment |
|  | PKD diet hungry | Is coffee bad for polycystic kidney disease? | polycystic kidney disease life expectancy |
|  | PKD diet recipes | What colour is urine when your kidneys are failing? | polycystic kidney disease causes |
|  | PKD paleo | Is corn bad for kidneys? | PKD keto diet |
|  | PKD diet list | Is quinoa bad for kidneys? | Polycystic kidney disease symptoms |
|  | PKD diet reddit | Can you drink alcohol with polycystic kidney disease? |  |
|  | PKD and diet | Are you born with polycystic kidney disease? |  |
|  |  | Can PKD skip a generation? |  |
|  |  | What does PKD pain feel like? |  |
|  |  | What fruit is good for kidneys? |  |
|  |  | What can I eat for breakfast with kidney disease? |  |
|  |  | Is there a genetic test for PKD? |  |
| PCKD and diet | CKD and diet | What foods should you avoid with polycystic kidney disease? | polycystic kidney disease diet recommendations |
|  | CKD and diet pdf | what is the best diet for polycystic kidney disease? | Polycystic kidney disease diet recipes |
|  | CKD and diet soda | What is the life expectancy of someone with polycystic kidney disease? | What foods should you avoid with polycystic kidney disease? |
|  | CKD and diet coke | is coffee bad for polycystic kidney disease? | polycystic kidney disease in children |
|  | Polycystic kidney disease and diet |  | polycystic kidney disease treatment |
|  | PKD diet |  | polycystic kidney disease life expectancy |
|  | stage 3 CKD and diet |  | polycystic kidney disease causes |
|  | CKD and diabetes diet |  | Polycystic kidney disease symptoms |
|  | CKD and keto diet |  |  |
|  | CKD and vegan diet |  |  |
| PKD and Nutrition | CKD and nutrition | What should I eat if I have PKD? | polycystic kidney disease diet recommendations |
|  | CKD and parenteral nutrition | What foods should you avoid with polycystic kidney disease? * | Polycystic kidney disease diet recipes |
|  |  | What is PKD belly? | What foods should you avoid with polycystic kidney disease? |
|  |  | What is the difference between CKD and PKD? | polycystic kidney disease in children |
|  |  |  | polycystic kidney disease treatment |
|  |  |  | polycystic kidney disease life expectancy |
|  |  |  | polycystic kidney disease causes |
|  |  |  | Polycystic kidney disease symptoms |
| PCKD and nutrition | CKD and nutrition | what is the best diet for polycystic kidney disease? | polycystic kidney disease diet recommendations |
|  | polycystic kidney disease and nutrition | What foods should you avoid with polycystic kidney disease? | Polycystic kidney disease diet recipes |
|  | CKD and parenteral nutrition | what is the life expectancy of someone with polycystic kidney disease? | What foods should you avoid with polycystic kidney disease? |
|  |  | What is the difference between CKD and PKD? | polycystic kidney disease in children |
|  |  |  | polycystic kidney disease treatment |
|  |  |  | polycystic kidney disease life expectancy |
|  |  |  | polycystic kidney disease causes |
|  |  |  | Polycystic kidney disease symptoms |
| PKD and food | PKD and food allergies | What should I eat if I have PKD?* | What foods should you avoid with polycystic kidney disease? * |
|  | PKD food to avoid | What foods should you avoid with polycystic kidney disease? * | polycystic kidney disease diet recommendations* |
|  | PKD food meaning | What does PKD stand for on food | Polycystic kidney disease diet recipes* |
|  | PKD food list* | what is the life expectancy of someone with polycystic kidney disease?* | kidney cysts foods to avoid |
|  | PKD food | Are you born with polycystic kidney disease? | PKD diet chart* |
|  | PKD food label | What is PKD belly?* | polycystic kidney disease treatment* |
|  | PKD food restriction* |  | polycystic kidney disease life expectancy* |
|  | PKD foods to eat* |  | PKD keto diet* |
| PCKD food | CKD foods to avoid* | What foods should you avoid with polycystic kidney disease? * | polycystic kidney disease diet recommendations* |
|  | CKD food | what is the life expectancy of someone with polycystic kidney disease?* | Polycystic kidney disease diet recipes* |
|  | CKD food list | Can you reverse polycystic kidney disease? | What foods should you avoid with polycystic kidney disease? * |
|  | CKD food pyramid | How do you treat polycystic kidney disease?* | polycystic kidney disease in children* |
|  | CKD food chart |  | polycystic kidney disease treatment* |
|  | CKD food diet* |  | polycystic kidney disease life expectancy* |
|  | CKD food for dogs |  | polycystic kidney disease causes* |
|  | CKD food restrictions |  | Polycystic kidney disease symptoms* |
|  | CKD foods you can eat |  |  |
| PCKD and food | CKD foods to avoid* | What foods should you avoid with polycystic kidney disease? * | What foods should you avoid with polycystic kidney disease? * |
|  | CKD and food* | what foods should I eat with PKD?* | polycystic kidney disease diet recommendations* |
|  | CKD and Chinese food | what is the life expectancy of someone with polycystic kidney disease?* | Polycystic kidney disease diet recipes* |
|  | CKD and fermented food | Is coffee bad for polycystic kidney disease? | kidney cysts foods to avoid* |
|  | CKD and Japanese food |  | PKD diet chart* |
|  |  |  | polycystic kidney disease treatment* |
|  |  |  | polycystic kidney disease life expectancy* |
|  |  |  | PKD keto diet* |
| PKD FAQ | PKD FAQ | Can PKD only affect one kidney? | polycystic kidney disease treatment* |
|  |  | what is the life expectancy of someone with polycystic kidney disease?* | polycystic kidney disease diagnosis |
|  |  | What should I eat if I have PKD?* | polycystic kidney disease diet* |
|  |  | Can you drink alcohol with polycystic kidney Disease? | polycystic kidney disease life expectancy* |
|  |  | Can PKD skip a generation? | Polycystic kidney disease causes* |
|  |  | What does PKD pain feel like? | Polycystic kidney disease in children* |
|  |  |  | polycystic kidney disease complication |
|  |  |  | polycystic kidney disease prognosis |
| PCKD FAQ | polycystic kidney disease FAQ* | What foods should you avoid with polycystic kidney disease? * | polycystic kidney disease treatment* |
|  |  | Can you die from polycystic kidney disease? | polycystic kidney disease diagnosis* |
|  |  | what is the life expectancy of someone with polycystic kidney disease?* | polycystic kidney disease diet* |
|  |  | Can PKD skip a generation? | polycystic kidney disease life expectancy* |
|  |  |  | Polycystic kidney disease causes* |
|  |  |  | Polycystic kidney disease in children* |
|  |  |  | polycystic kidney disease complication* |
|  |  |  | polycystic kidney disease prognosis* |

Legend: CKD: Chronic kidney disease ; FAQ: frequently asked question; PCKD Polycystic kidney disease; PKD Polycystic kidney disease
