# Supplementary material for: What are the information needs and concerns of individuals with Polycystic Kidney Disease? Results of an online survey using Facebook and social listening analysis
Source: BMC Nephrol. 2021 Jul 14;22:263. doi: 10.1186/s12882-021-02472-1 (PMC8281574; doi:10.1186/s12882-021-02472-1)
Supplement: Supplementary file 2 — Additional file 2: Supplementary Table S2. Summary of social listening analysis of frequently asked questions by individuals with PKD and their carers using Google ‘people questions ask’. [file 12882_2021_2472_MOESM2_ESM.docx]

Supplementary Table 2. Summary of social listening analysis content analysis of frequently asked questions by individuals with PKD and their carers using Google ‘people questions ask’.

| **Major categories** | **Examples of common questions included in this category** |
| --- | --- |
| PKD and Diet | General diet for people with PKD, specific diets for PKD, diet information related to stage of PKD, diet information for people undertaking any type of dialysis, diet information specifically for people undertaking peritoneal dialysis, diet information for people specifically undertaking haemodialysis, diet information for people after a nephrectomy, PKD specific recipes |
| PKD and nutrition | Supplements, how to eat to lose weight, reference to evidence-based nutrition guidelines, resources for people with PKD |
| PKD and food | Types of foods/beverages to avoid for people with PKD, types of foods/beverages to consume for people with PKD, reading food label for people with PKD, alcohol, protein-based food, miscellaneous food questions, caffeine |
| PKD FAQ | Medication, dialysis, nephrectomy, surgery, background information about PKD, diagnosis of PKD, Hospitals, nephrologist, Encouragement/support/humour, Grievance/frustration/anger, symptoms of PKD, information relating to renal function and stage, surgery complications, related medical conditions, unrelated medical conditions, children with PKD, physical activity, transplant, kidney donor, insurance, mental health, alternative therapies |

Legend: PKD Polycystic kidney disease; FAQ frequently asked question
