# Supplementary material for: What are the information needs and concerns of individuals with Polycystic Kidney Disease? Results of an online survey using Facebook and social listening analysis
Source: BMC Nephrol. 2021 Jul 14;22:263. doi: 10.1186/s12882-021-02472-1 (PMC8281574; doi:10.1186/s12882-021-02472-1)
Supplement: Supplementary file 3 — Additional file 3: Supplementary Table S3: Categorisation of all Facebook Groups posts. [file 12882_2021_2472_MOESM3_ESM.docx]

Supplementary Table 3. Categorisation of all Facebook Groups posts

| **Other FAQs** | **Frequency** | **Percent** | **Medical questions** | **Frequency** | **Percent** |
| --- | --- | --- | --- | --- | --- |
| Coping with PKD | 6 | 22.2 | Anaemia | 2 | 1.2 |
| Insurance | 8 | 29.6 | Aneurism | 2 | 1.2 |
| Nephrologist Questions | 2 | 7.4 | Blood in urine | 3 | 1.8 |
| Physical Activity | 1 | 3.7 | Bone Marrow Suppression | 1 | .6 |
| Physical Activity and Lifestyle | 1 | 3.7 | Creatine levels | 1 | .6 |
| Physical Activity | 1 | 3.7 | Cyst | 1 | .6 |
| Physical activity and creatine | 1 | 3.7 | Cyst burst , Medication | 1 | .6 |
| PKD Category | 1 | 3.7 | Cyst burst, medication | 1 | .6 |
| PKD Information | 1 | 3.7 | Cyst Haemorrhaging | 1 | .6 |
| Resources | 1 | 3.7 | Dialysis | 2 | 1.2 |
| weight loss | 1 | 3.7 | Diagnosis | 2 | 1.2 |
| Weight loss | 1 | 3.7 | Diagnosis and children | 4 | 2.4 |
| Total | 27 | 100.0 | Diagnosis, Symptoms, GFR, Surgery | 1 | .6 |
|  |  |  | Dialysis | 11 | 6.7 |
|  |  |  | Dialysis and Dehydration | 1 | .6 |
|  |  |  | Dialysis and Diagnosis | 1 | .6 |
|  |  |  | Dialysis and fatigue | 1 | .6 |
|  |  |  | Dialysis and transplant | 1 | .6 |
|  |  |  | Dialysis, fatigue | 1 | .6 |
|  |  |  | Fatigue | 4 | 2.4 |
|  |  |  | Fatigue and Pain | 1 | .6 |
|  |  |  | GFR | 3 | 1.8 |
|  |  |  | General medical | 22 | 13.3 |
|  |  |  | Heart Condition | 1 | .6 |
|  |  |  | Iron | 1 | .6 |
|  |  |  | Kidney Function, Kidney Size | 1 | .6 |
|  |  |  | Kidney Size | 4 | 2.4 |
|  |  |  | Medication | 17 | 10.3 |
|  |  |  | Medication, Dialysis, GFR | 1 | .6 |
|  |  |  | Pain | 14 | 8.5 |
|  |  |  | Pain and alcohol | 1 | .6 |
|  |  |  | Pain, cyst burst | 1 | .6 |
|  |  |  | Pain, Dialysis | 1 | .6 |
|  |  |  | Pancreatic cysts, treatment | 1 | .6 |
|  |  |  | Parvovirus | 1 | .6 |
|  |  |  | Pelvic Floor rehab, treatments | 1 | .6 |
|  |  |  | PLD | 3 | 1.8 |
|  |  |  | PLD, medication and high cholesterol | 1 | .6 |
|  |  |  | Sponge Kidneys | 1 | .6 |
|  |  |  | Supplements | 4 | 2.4 |
|  |  |  | Surgery | 8 | 4.8 |
|  |  |  | Surgery and Pain | 1 | .6 |
|  |  |  | Surgery, low blood pressure | 1 | .6 |
|  |  |  | Surgery | 1 | .6 |
|  |  |  | Symptoms | 12 | 7.3 |
|  |  |  | Symptoms and PLD | 1 | .6 |
|  |  |  | Transplant | 12 | 7.3 |
|  |  |  | Transplant and Blood in urine | 1 | .6 |
|  |  |  | Transplant and Medication | 1 | .6 |
|  |  |  | Transplant and Surgery | 1 | .6 |
|  |  |  | Treatments | 3 | 1.8 |
|  |  |  | UTI, Kidney function | 1 | .6 |
|  |  |  | Total | 165 | 100.0 |

**Categorisation of Diet and Nutrition related posts**

| Nutrition related | Frequency | Percent | Diet related | Frequency | Percent |
| --- | --- | --- | --- | --- | --- |
| how to eat to lose weight | 5 | 7.0 | diet dialysis | 1 | .8 |
| reference to evidence based nutrition guidelines | 2 | 2.8 | diet for stage of PKD | 3 | 2.5 |
| reference to evidence based information for people with PKD | 1 | 1.4 | general diet for people with PKD | 1 | .8 |
| resources for people with PKD | 4 | 5.6 | general diet for people with PKD | 10 | 8.3 |
| Supplements | 54 | 76.0 | general diet for PKD | 1 | .8 |
| Total | 5 | 7.0 | general diet for PKD | 2 | 1.7 |
|  | 71 | 100.0 | general diets for PKD/specific diet for people with PKD | 1 | .8 |
|  |  |  | general diet for PKD | 1 | .8 |
|  |  |  | PKD specific recipes | 2 | 1.7 |
|  |  |  | specific diet for people with PKD | 2 | 1.7 |
|  |  |  | specific diet for PKD | 12 | 10.0 |
|  |  |  | specific diet for PKD/diet information related to stage of PKD | 1 | .8 |
|  |  |  | specific diets for people with PKD | 2 | 1.7 |
|  |  |  | specific diets for people with PKD | 2 | 1.7 |
|  |  |  | specific diets for PKD | 1 | .8 |
|  |  |  | specific diets for PKD | 69 | 57.5 |
|  |  |  | specific diets for PKD/diet information related to stage of PKD | 4 | 3.3 |
|  |  |  | specific diets for PKD/PKD specific recipes | 5 | 4.2 |
|  |  |  | Total | 120 | 100.0 |

Categorisation of food and other related posts

| Other related posts | Frequency | Percent | Food related posts | Frequency | Percent |
| --- | --- | --- | --- | --- | --- |
| mental health/encouragement/support/humour | 1 | .2 |  | 3 | 3.8 |
| alternative therapies | 1 | .2 | alcohol | 1 | 1.3 |
| alternative therapies | 7 | 1.1 | caffeine | 10 | 12.5 |
| alternative therapies/dialysis | 1 | .2 | caffeine/miscellaneous food questions | 1 | 1.3 |
| alternative therapies/information relating to renal function and stage | 3 | .5 | caffeine/types of foods to avoid for people with PKD | 1 | 1.3 |
| alternative therapies/symptoms of PKD | 1 | .2 | foods to avoid eating for people with PKD | 1 | 1.3 |
| alternative therapies | 1 | .2 | miscellaneous food questions | 29 | 36.3 |
| background information about PKD | 1 | .2 | miscellaneous food questions | 1 | 1.3 |
| background information about PKD | 9 | 1.4 | Protein -based foods | 1 | 1.3 |
| Background information about PKD | 1 | .2 | protein based foods | 1 | 1.3 |
| background information about PKD/diagnosis of PKD | 1 | .2 | protein-based food | 3 | 3.8 |
| background information about PKD/diagnosis of PKD | 1 | .2 | protein-based foods | 2 | 2.5 |
| background information about PKD/encouragement/support/humour | 1 | .2 | type of food to eat for people with PKD | 1 | 1.3 |
| background information about PKD/information relating to renal function and stage | 2 | .3 | types of food to avoid for people with PKD | 4 | 5.0 |
| background information about PKD/encouragement/support/humour | 1 | .2 | types of food to eat for people with PKD | 1 | 1.3 |
| background information about PKD/symptoms of PKD | 1 | .2 | Types of food to eat for people with PKD | 4 | 5.0 |
| background information on PKD | 1 | .2 | types of food to eat for people with PKD/types of food to avoid for people with PKD | 1 | 1.3 |
| Background information on PKD | 5 | .8 | types of foods for people to eat with PKD | 1 | 1.3 |
| background information relating to PKD | 2 | .3 | types of foods to avoid for people with PKD | 1 | 1.3 |
| background information about PKD | 1 | .2 | types of foods to avoid for people with PKD | 4 | 5.0 |
| Children and PKD | 1 | .2 | types of foods to eat for people with PKD | 7 | 8.8 |
| children with PKD | 2 | .3 | types of foods to eat for people with PKD/types of foods to avoid for people with PKD | 1 | 1.3 |
| children with PKD | 3 | .5 | types of foods to eat for people with PKD/types of foods to avoid for people with PKD | 1 | 1.3 |
| children with PKD/background information about PKD/diagnosis of PKD | 1 | .2 | Total | 80 | 100.0 |
| Children with PKD/diagnosis | 2 | .3 |  |  |  |
| children with PKD/diagnosis of PKD | 1 | .2 |  |  |  |
| children with PKD/diagnosis of PKD/encouragement/support/humour | 1 | .2 |  |  |  |
| Children with PKD/diagnosis | 1 | .2 |  |  |  |
| children with PKD/dialysis/grievance/frustration/anger | 1 | .2 |  |  |  |
| children with PKD/encouragement/support/humour | 1 | .2 |  |  |  |
| children with PKD/kidney donor/encouragement/support/humour | 1 | .2 |  |  |  |
| children with PKD/nephrologist | 1 | .2 |  |  |  |
| children with PKD/symptoms of PKD | 1 | .2 |  |  |  |
| diagnosis of PKD | 2 | .3 |  |  |  |
| diagnosis of PKD/background information about PKD | 1 | .2 |  |  |  |
| diagnosis of PKD/encouragement/support/humour | 1 | .2 |  |  |  |
| diagnosis of PKD/symptoms of PKD/medication | 1 | .2 |  |  |  |
| dialysis | 12 | 1.9 |  |  |  |
| Dialysis | 1 | .2 |  |  |  |
| dialysis/ information relating to renal function and stage | 1 | .2 |  |  |  |
| dialysis/encouragement/support/humour | 5 | .8 |  |  |  |
| dialysis/grievance/frustration/anger | 1 | .2 |  |  |  |
| dialysis/information relating to renal function and stage | 2 | .3 |  |  |  |
| dialysis/support/encouragement/humour | 1 | .2 |  |  |  |
| dialysis/surgery | 1 | .2 |  |  |  |
| dialysis/symptoms of PKD | 1 | .2 |  |  |  |
| dialysis/transplant | 1 | .2 |  |  |  |
| dialysis/transplant/grievance/frustration/anger | 1 | .2 |  |  |  |
| encouragement | 1 | .2 |  |  |  |
| Encouragement, support/humour | 1 | .2 |  |  |  |
| encouragement/supper/humour | 1 | .2 |  |  |  |
| encouragement/support/humour | 1 | .2 |  |  |  |
| encouragement/support/humour | 92 | 14.5 |  |  |  |
| Encouragement/support/humour | 12 | 1.9 |  |  |  |
| encouragement/support/humour/dialysis/transplant | 1 | .2 |  |  |  |
| encouragement/support/humour/medication | 1 | .2 |  |  |  |
| Encouragement/support/humour/nephrologist | 1 | .2 |  |  |  |
| Encouragement/support/humour/Transplant | 1 | .2 |  |  |  |
| encouragement/support/humour/transplant/dialysis | 1 | .2 |  |  |  |
| encouragement/support/humour/unrelated medical condition | 1 | .2 |  |  |  |
| encouragement/support/PKD | 1 | .2 |  |  |  |
| encouragement/support/humour | 1 | .2 |  |  |  |
| encouragement/support/humour | 1 | .2 |  |  |  |
| grievance/frustration/anger | 12 | 1.9 |  |  |  |
| Grievance/frustration/anger | 4 | .6 |  |  |  |
| grievance/frustration/anger/children with PKD | 1 | .2 |  |  |  |
| Grievance/frustration/anger/dialysis | 1 | .2 |  |  |  |
| grievance/frustration/anger/information relating to renal function and stage | 1 | .2 |  |  |  |
| grievance/frustration/anger | 1 | .2 |  |  |  |
| grievance/frustration/anger | 3 | .5 |  |  |  |
| hospital | 1 | .2 |  |  |  |
| hospitals | 1 | .2 |  |  |  |
| hospitals/transplant | 1 | .2 |  |  |  |
| information on renal function and stage/surgery complications | 1 | .2 |  |  |  |
| information relating to renal function and stage/dialysis | 1 | .2 |  |  |  |
| information relating to renal function and stage | 24 | 3.8 |  |  |  |
| Information relating to renal function and stage | 1 | .2 |  |  |  |
| information relating to renal function and stage/background information about PKD | 1 | .2 |  |  |  |
| information relating to renal function and stage/encouragement/support/humour | 2 | .3 |  |  |  |
| Information relating to renal function and stage/Encouragement/support/humour | 2 | .3 |  |  |  |
| information relating to renal function and stage/encouragement/support/humour/alternative therapies | 1 | .2 |  |  |  |
| information relating to renal function and stage/grievance/frustration/anger | 1 | .2 |  |  |  |
| information relating to renal function and stage/mental health/grievance/frustration/anger/transplant | 1 | .2 |  |  |  |
| Information relating to renal function and stage/Physical activity | 1 | .2 |  |  |  |
| information relating to renal function and stage/symptoms of PKD | 4 | .6 |  |  |  |
| information relating to renal function and stage/transplant | 1 | .2 |  |  |  |
| information relating to renal function and stage/unrelated medical conditions | 1 | .2 |  |  |  |
| Information relation to renal function and stage | 1 | .2 |  |  |  |
| insurance | 6 | .9 |  |  |  |
| Insurance | 3 | .5 |  |  |  |
| insurance/children with PKD/diagnosis of PKD | 1 | .2 |  |  |  |
| kidney donor | 1 | .2 |  |  |  |
| Kidney donor | 1 | .2 |  |  |  |
| kidney donor/children with PKD/encouragement/support/humour | 1 | .2 |  |  |  |
| kidney donor/encouragement/support/humour | 18 | 2.8 |  |  |  |
| Kidney donor/encouragement/support/humour | 1 | .2 |  |  |  |
| kidney donor/encouragement/support/PKD | 1 | .2 |  |  |  |
| kidney donor/grievance/frustration/anger | 1 | .2 |  |  |  |
| Kidney donor/transplant/encouragement/support/humour | 1 | .2 |  |  |  |
| kidney donor/transplant/encouragement/support/humour/hospital | 1 | .2 |  |  |  |
| kidney donors | 1 | .2 |  |  |  |
| medication | 31 | 4.9 |  |  |  |
| Medication | 4 | .6 |  |  |  |
| medication/alternative therapies/symptoms of PKD | 1 | .2 |  |  |  |
| medication/encouragement/support/humour | 1 | .2 |  |  |  |
| medication/grievance/frustration/anger | 1 | .2 |  |  |  |
| medication/insurance | 1 | .2 |  |  |  |
| medication/mental health | 1 | .2 |  |  |  |
| medication/nephrologist | 1 | .2 |  |  |  |
| medication/related medical conditions | 2 | .3 |  |  |  |
| medication/symptoms of PKD | 1 | .2 |  |  |  |
| medication/symptoms of PKD/information relating to renal function and stage | 1 | .2 |  |  |  |
| medication/transplant | 1 | .2 |  |  |  |
| medication/unrelated medical conditions | 2 | .3 |  |  |  |
| medications | 3 | .5 |  |  |  |
| mental health | 1 | .2 |  |  |  |
| mental health/grievance/frustration/anger | 1 | .2 |  |  |  |
| nephrectomy | 3 | .5 |  |  |  |
| Nephrectomy | 1 | .2 |  |  |  |
| Nephrectomy/dialysis | 1 | .2 |  |  |  |
| Nephrectomy/Encouragement/support/humour | 1 | .2 |  |  |  |
| nephrectomy/surgery complication | 1 | .2 |  |  |  |
| nephrologist | 5 | .8 |  |  |  |
| Nephrologist | 2 | .3 |  |  |  |
| nephrologist/information about renal function and stage | 1 | .2 |  |  |  |
| nephrologist/information relating to renal function and stage | 1 | .2 |  |  |  |
| nephrologist/information relating to renal function and stage/symptoms of PKD | 1 | .2 |  |  |  |
| Pain/Grievance/frustration/anger | 1 | .2 |  |  |  |
| physical activity | 3 | .5 |  |  |  |
| physical activity/encouragement/support/humour | 1 | .2 |  |  |  |
| related medical conditions/information relating to renal function and stage | 1 | .2 |  |  |  |
| related medical conditions/surgery | 1 | .2 |  |  |  |
| related medical condition/dialysis/transplant | 1 | .2 |  |  |  |
| related medical condition/grievance/frustration/anger | 1 | .2 |  |  |  |
| related medical conditions | 20 | 3.2 |  |  |  |
| Related medical conditions | 6 | .9 |  |  |  |
| related medical conditions/encouragement/support/humour | 3 | .5 |  |  |  |
| related medical conditions/grievance/frustration/anger | 4 | .6 |  |  |  |
| related medical conditions/medication | 1 | .2 |  |  |  |
| related medical conditions/surgery | 2 | .3 |  |  |  |
| Related medical conditions/symptoms of PKD | 1 | .2 |  |  |  |
| related medical conditions/transplant | 1 | .2 |  |  |  |
| Related medical conditions/transplant/dialysis | 1 | .2 |  |  |  |
| related medication conditions/transplant | 1 | .2 |  |  |  |
| surgery | 6 | .9 |  |  |  |
| Surgery | 2 | .3 |  |  |  |
| surgery complications | 1 | .2 |  |  |  |
| Surgery/Encouragement/support/humour | 1 | .2 |  |  |  |
| surgery/unrelated medical conditions | 1 | .2 |  |  |  |
| symptoms of PKD | 1 | .2 |  |  |  |
| symptoms of PKD/transplant/information relating to renal function and stage | 1 | .2 |  |  |  |
| symptoms of PKD | 33 | 5.2 |  |  |  |
| symptoms of PKD | 13 | 2.1 |  |  |  |
| Symptoms of PKD | 6 | .9 |  |  |  |
| symptoms of PKD/background information about PKD | 1 | .2 |  |  |  |
| Symptoms of PKD/background information about PKD | 1 | .2 |  |  |  |
| symptoms of PKD/dialysis | 1 | .2 |  |  |  |
| Symptoms of PKD/dialysis | 1 | .2 |  |  |  |
| symptoms of PKD/mental health | 1 | .2 |  |  |  |
| symptoms of PKD/encouragement/support/humour | 3 | .5 |  |  |  |
| Symptoms of PKD/Encouragement/support/humour | 1 | .2 |  |  |  |
| symptoms of PKD/grievance/frustration/anger | 6 | .9 |  |  |  |
| symptoms of PKD/grievance/frustration/anger | 4 | .6 |  |  |  |
| Symptoms of PKD/Grievance/frustration/anger | 1 | .2 |  |  |  |
| symptoms of PKD/grievance/frustration/anger/information relating to renal function and stage | 1 | .2 |  |  |  |
| symptoms of PKD/grievance/frustration/pain | 1 | .2 |  |  |  |
| symptoms of PKD/information relating to renal function and stage | 4 | .6 |  |  |  |
| symptoms of PKD/information relating to renal function and stage | 3 | .5 |  |  |  |
| symptoms of PKD/information relating to renal function and stage/dialysis/transplant | 1 | .2 |  |  |  |
| symptoms of PKD/mental health | 1 | .2 |  |  |  |
| symptoms of PKD/nephrologist | 1 | .2 |  |  |  |
| symptoms of PKD/surgery | 1 | .2 |  |  |  |
| symptoms of PKD/grievance/frustration/anger | 1 | .2 |  |  |  |
| symptoms of PKD | 1 | .2 |  |  |  |
| Symptoms of PKD | 2 | .3 |  |  |  |
| Symptoms of PKD/in relation to renal function and stage | 1 | .2 |  |  |  |
| symptoms of PKD/information relating to renal function and stage/diagnosis of PKD | 1 | .2 |  |  |  |
| Symptoms of PKD/support/encouragement/humour | 1 | .2 |  |  |  |
| transplant | 17 | 2.7 |  |  |  |
| Transplant | 6 | .9 |  |  |  |
| transplant/dialysis | 1 | .2 |  |  |  |
| transplant/encouragement/support/humour | 2 | .3 |  |  |  |
| Transplant/Encouragement/support/humour | 1 | .2 |  |  |  |
| transplant/encouragement/support/humour/unrelated medical conditions | 1 | .2 |  |  |  |
| transplant/hospital | 1 | .2 |  |  |  |
| transplant/insurance | 1 | .2 |  |  |  |
| Transplant/insurance | 1 | .2 |  |  |  |
| Transplant/Kidney donation | 1 | .2 |  |  |  |

Legend: PKD Polycystic kidney disease; FAQ frequently asked question; GFR glomerular filtration rate; UTI urinary tract infection; PLD polycystic liver disease
